# The Lady and the Plants: Two Notions of Teleology in Agnes Arber’s Philosophy of Plants

**DOI:** 10.1007/s10739-024-09793-5

**Published:** 2025-01-07

**Authors:** Vera Maximilia Straetmanns

**Affiliations:** Ruhr University Bochum, Bochum, Germany

**Keywords:** Agnes Arber, Teleology, Plant philosophy, Morphology, Evo-devo

## Abstract

Agnes Arber (1879–1960) was a British plant morphologist, historian of botany, and philosopher of biology. Though now largely forgotten, her work offers valuable insights into morphological as well as philosophical issues. This paper focuses on Arber’s work on teleology in plants. After providing a brief overview of her life and distinct style of work, two notions of teleology are presented, which become apparent in Arber’s morphological and philosophical work. The first notion, labeled *final teleology*, is based on Aristotle’s final cause and deals with adaptation-based explanations in biology. The second is labeled *formal teleology*. It is grounded in the Aristotelian formal cause and deals with the inherent directiveness of developing structures and the actualization of potentialities in organisms and their parts. Whereas Arber showed a reserved and skeptical attitude towards final teleology, she was very sympathetic to formal teleology, building her general morphological framework on it. Two examples from Arber’s work are then given, which illustrate how formal teleology informed her theorizing: the partial-shoot theory of the leaf, and parallelism in evolution as a counter-proposal to natural selection. Finally, Arber’s teleological interpretation of plant morphology is historically contextualized and connected to recent research developments in evolutionary biology and plant morphology.

## Botany and Teleology in the Historiography of Biology

Though much work has been done in recent years on the historiography of botany, albeit still to a much lesser degree than on the historiography of zoology, there are many areas that lack in-depth consideration. While some subjects have been treated in a comparatively extensive manner, such as the emergence of botany as a science during the 18th century and its subsequent professionalization, the suppression of female voices, as well as botany’s role in colonialism, and its influence on the development of the “evolutionary synthesis,” the history of theoretical and philosophical reasoning about plants in particular has received less scholarly attention.^1^ Some historical work has recently been done on concepts and theories of plant behavior and intelligence as well as plant sexuality, but an area that has seldom been focused on is the way plant researchers have advanced and influenced ideas on teleology in the natural world.^2^

Teleology occupied the thinking of natural philosophers since antiquity, and has never left biology or the philosophy of biology. Especially in recent decades, teleological explanations in biology have been reconsidered and are often viewed as indispensable (Illetterati and Michelini 2013). Considering plant sciences in particular, teleological considerations importantly shape current discussions, such as those surrounding potential agential capabilities in plants.^3^ Also within the vitalism-mechanism debate that took place in the late 19th and early 20th century biology and philosophy of biology, teleology was a hotly debated topic (Baedke et al. 2024). While vitalists highlighted the purposefulness in developing organisms, mechanists strongly rejected teleological explanations in biology. From this quarrel arose the movement of organicism or “organism-centered biology” during the interwar period.^4^ Scholars in this movement wanted to find a third way or middle ground between mechanism and vitalism which for many included the intent to save teleology as a guiding and necessary principle in understanding living organisms while at the same time getting rid of vitalistic notions in biology (Baedke 2019).

But even though teleology was a major issue in these debates, historians of biology have so far not investigated teleological accounts in the early 20th century very extensively, and botany is especially a black box in this regard. This is why this paper spotlights the teleological considerations of an organicist writer, active in the first half of the 20th century: British plant morphologist Agnes Arber (1879–1960).^5^ Arber occupied a unique position, bringing together her expertise from the fields of history of botany, plant morphology, and philosophy of biology. Furthermore, she held unusual interests and belonged to different sets of groups we might class as minorities: she was a woman in academia in as well as a single mother; she was a philosopher of biology who mainly reasoned about plants instead of animals; and she was a natural scientist with nuanced positions on teleology and metaphysics. Arber also possessed broad knowledge of philosophy, literature, and art. This exceptional combination of interdisciplinary influences and outlooks “fostered the deep analysis of philosophical issues in biology that makes her work worthy of continued attention today“ (Flannery 2003, p. 282).

There has been some scholarly work conducted on Arber and her botanical as well as philosophical ideas. After her death in 1960, several obituaries were published the most comprehensive of which was by Hugh Hamshaw Thomas (1885–1962), a distinguished British plant morphologist and paleobotanist (Thomas 1960a).^6^ More recent biographically focused papers about Arber are available from Packer (1997) and Schmid (2001). Other publications on Arber examine specific books written by her, draw comparisons to other scholars working on plant morphology, or apply some of Arber’s theories and ideas to current research in plant sciences.^7^ Some papers exist that focus on her positions concerning the philosophy of biology (Sattler 2001; Bruce 2014; Yilmaz 2024), but there are still considerable gaps in the scholarship about Arber, one being her unique understanding of teleology in the natural world. The goal of this paper is thus to offer a contribution towards filling this gap. Moreover, it intends to show how Arber’s innovative approach can be an inspiration for current philosophical treatments of plants and can enrich the historiography of botany through exploration of its philosophical dimensions.

The next section gives an overview of Arber’s life and work by drawing on archival material from the Hunt Institute for Botanical Documentation, Pittsburgh, and from the archive of D’Arcy Wentworth Thompson at the University of St. Andrews. In the following section, two distinct notions of teleology that characterize Arber’s standpoint are identified and categorized as *formal* and *final* teleology, referring to Aristotle’s formal and final cause, respectively. While this terminological distinction is not stated in Arber’s original works, it is introduced here as a framework with the intention to reconstruct and better understand Arber’s lines of thought. I argue that the notion of formal teleology was the more important and relevant one for Arber and that most of her morphological and philosophical work was directly or indirectly informed by it. The next two sections illustrate Arber’s use and understanding of formal teleology in the context of two of her theories: the partial-shoot theory of the leaf and the idea of parallelism in evolution. The final section contextualizes Arber’s teleological interpretation of plant morphology within biology and philosophy of the early to mid-20th century. Furthermore, it points to some present-day fields of research, like plant morphology and evolutionary biology that could benefit from a renewed interest in Arber’s work. In this context, the relation of some of Arber’s ideas to the field of evolutionary developmental biology is highlighted.

## The Life and Work of Agnes Arber

**Fig. 1:**
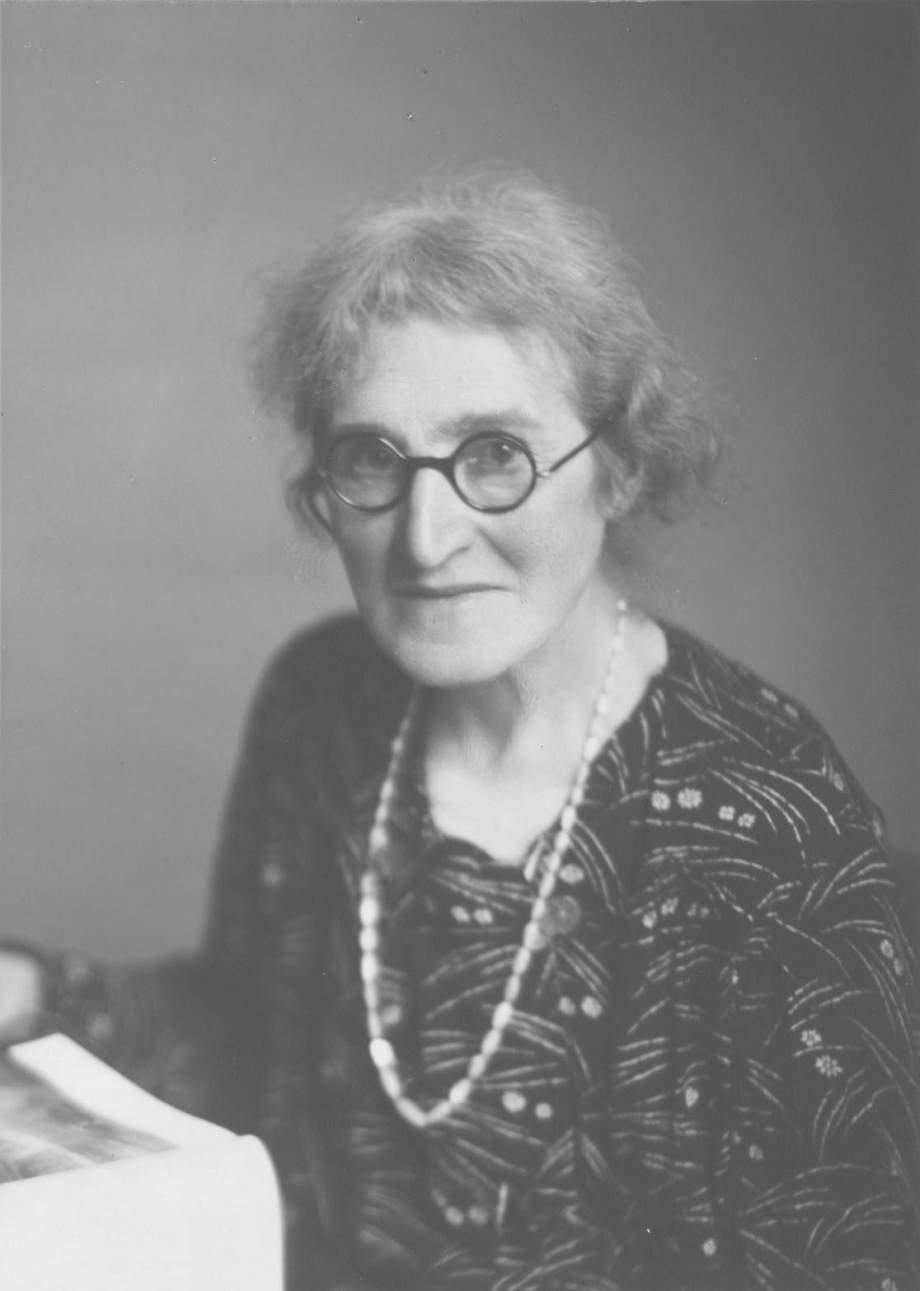
Agnes Arber 1946. Official photograph taken on behalf of the Royal Society on her election as a Fellow. Photographed by Walter Stoneman, 1946 © Godfrey Argent Studio, courtesy of the Royal Society.

Agnes Arber, née Robertson, was born on 23 February 1879 and died on 22 March 1960. Her interest in plants developed early in life and at only the age of seven she created her first herbarium.^8^ Arber later studied at the University College London and the University of Cambridge, and received her doctoral degree from University College London in 1905 for research on the reproductive morphology of the gymnosperm *Torreya californi*. After working as a research assistant for the plant morphologist Ethel Sargant (1863–1918) and as researcher and lecturer at the University College London, she spent the rest of her life in Cambridge where she worked as a researcher and writer. In her lifetime, she received notable recognition for her contributions to botany. In 1946, for example, Arber was elected a Fellow of the Royal Society of London (Fig. 1) as the first female botanist and the third woman overall, and many considered her the leading woman botanist of her time. According to her obituary in the *Proceedings of the Linnean Society of London* (she had become a Fellow in 1908), for example, Arber was considered as “the lady of botany” by many of her contemporaries (Anonymous 1961, p. 128).

Until 1927, Arber worked in the Balfour Biological Laboratory for Women at Newnham College. After this was closed down, she set up a laboratory in her home in Cambridge and performed her morphological research in these modest facilities.^9^ This research included the analysis of plants in different developmental stages under a dissecting microscope, as well as preparing and analyzing cross sections of plant tissue. Furthermore, Arber observed and described the morphology of plants growing in nature and also kept plants at home to study their growth and development. She worked alone, which meant that she carried out all technical aspects of her research herself, including assembling results, developing theories, and publishing papers. Her work also involved botanical illustrations as she was a very skilled draftswoman. Her father and her sister were painters and her father began giving Arber art lessons at the age of three, which she mentioned in a letter to Scottish biologist and polymath D’Arcy Wentworth Thompson (1860–1948), a long-standing correspondent.^10^ Most of her articles and books contain illustrations that she created herself. She was even commissioned by Thompson, who was very impressed by her skills, to do an illustration for his second edition of *On Growth and Form* (Thompson 1945).^11^

Arber seems to have adopted her independent style of research from her mentor and later friend Ethel Sargant, and passed it on to her daughter Muriel who worked as a geologist and a high school teacher. British botanist William T. Stearn (1911-2001), who was a longtime friend of Arber, reported that she told students in 1926: “[T]he concentration of mind necessary for independent thought is far more easily achieved in a place where one can get a generous measure of solitude than in a populous laboratory where people are incessantly running in and out, and in Ethel Sargant’s words—‘Independence is the essence of research’” (Stearn 1960, p. 263). Despite this solitary way of working, Schmid emphasizes that Arber has been wrongly described as a “recluse.”^12^ He cites several contemporaries who called her “gracious” and “always available to help colleagues” (Schmid 2001, p. 1119).

One disadvantage of working from home, however, was that it meant less regular contact with other biologists. For much of her scientific career, Arber had no day-to-day contact with students or other professors. However, she wrote and received many letters and was in contact with botanists in different countries like Britain, the USA, India, Germany, and the Soviet Union.^13^ She discussed articles by other scientists and also received feedback from them. Flannery points out that, even though Arber was perhaps missing the opportunity of casual conversation about her research with colleagues and students, a more traditional way of being a researcher “might also have given her less time for deep thought, so in the end, it might have made her contribution to science and the philosophy of science less noteworthy” (Flannery 2003, p. 285).

Arber wrote several books and over 90 scientific papers. Her publications are largely concerned with botanical subjects, but within them she covered a broad range of topics. Her first book was *Herbals: Their Origin and Evolution*, originally published in 1912, in which she traced the history of botanical herbals between 1470 and 1670 (Arber 1912). Later, she nurtured her interest in the history of botany and published papers about botanists from the Early Modern period like Robert Sharrock, Nehemiah Grew, and Marcello Malpighi (Arber 1942, 1960). Arber wrote fundamental morphological studies of three plant groups: water plants (1920), monocotyledons (1925) and Gramineae (1934). In all of them, she gave detailed descriptions of a vast number of species and their morphological and physiological features, but she also committed time to thinking about general problems of theoretical biology like individuality (Arber 1934, Chap. 10), natural selection (Arber 1920, Chap. 27), and parallelism in evolution (Arber 1925, Chap. 10). Her last two books transcended the field of biology and contained more general philosophical thoughts. *The Mind and the Eye* (1954) dealt with the standpoint of biologists and how they could philosophically reflect on their work, and *The Manifold and the One* (1957) discussed the phenomenon of mysticism and the relationship between unity and multiplicity in the natural world. This transition towards more theoretical issues coincided with Arber withdrawing from active morphological research during the Second World War.

Arber’s interest in philosophy, however, had started long before this time, possibly in her youth as her school education had a broad focus on the study of Classics, so she was familiar with the works of Aristotle, Plato, and the Neoplatonists. She was also well-versed in medieval and later philosophers and “read Dante as much as Shakespeare” (Feola 2019, p. 519). As Arber wrote to the botanist B. C. Sharman in 1939, one part of the research for her 1950 book *The Natural Philosophy of Plant Form* was “struggling with as much philosophy as I can grasp (alas that it is little) to try to clear up my approach to the subject”—with the subject being a consideration of “the morphology of flowering plants on general lines.”^14^*The Natural Philosophy of Plant Form* combined historical analyses, philosophical insights, theoretical considerations, and detailed descriptions of plant morphology and was, according to one recent account, “arguably […] her most important book” (Schmid 2001, p. 1107). In it, Arber displayed her conviction of the “vital necessity of a linkage between morphological and philosophic thought.” Through the theoretical and conceptual analysis of problems in plant morphology, she hoped to find the means for a “synthesis of various theories that are, from the standpoint of analytical science, irreconcilable” (Arber 1950, p. vii). This book, as well as several of her other works, also showed the influence that the botanical writings of Johann Wolfgang von Goethe (1749–1832) had on her. Arber encountered Goethe’s scientific work during her time in college (Feola 2019), and produced a translation of his *Versuch die Metamorphose der Pflanze zu erklären* during the summer of 1932,^15^ which was published together with an introduction by Arber in 1946. Arber called Goethe “a botanist of genius” (Arber 1961, p. 999) and felt that she had “a really clear notion of his thought (as distinct from what later poeple [sic] *think* he thought).”^16^ However, she also made critical remarks about his “amateur pursuit of science” (Arber 1946, p. 77). And while being “influenced by idealism,” Arber, in contrast to Goethe, always made sure to ground her philosophy in the practice of science and to give evidence for her theories from direct observation and through citing the work of others (Flannery 2003, p. 287).

With this background information about Arber’s life, work, and influences in mind, we can now turn to her specific approach to teleology in the natural world. For this, let us first see where in her works and in what manner she wrote about teleology.

### Arber’s Two Notions of Teleology

Remarks about teleological positions and related concepts are scattered throughout many of Arber’s earlier works. In the last chapter of her 1950 book, *The Natural Philosophy of Plant Form*, however, she explicitly tackled the topic of teleology in relation to biological research. Here, Arber sought to show how the teleological approach to plant morphology could be synthesized with the mechanical one.^17^ In the mechanical approach, organisms were basically conceptualized as elaborate machines and the focus of research lay on the collection of quantifiable data, as well as on the analysis of confined structures or processes. This attitude had been brought into botany by researchers like Matthias Jacob Schleiden (1804–1881), Julius Sachs (1832–1897) or Wilhelm Pfeffer (1845–1920) who advocated for an experimentally driven, inductive botany with plant physiology as its main focus and the aim to find the physico-chemical causes of plant processes (Jahn 1990, 2004). The contrasting approach, which Arber characterized as *teleological* but sometimes also called *morphological*, and which for her went back to Goethe, was mainly represented in botany by Wilhelm Troll (1897–1978) and his followers during Arber’s lifetime (see Levit and Meister 2006; Rieppel 2011).^18^ This approach took the whole organism into account rather than analyzing isolated structures or processes and it granted directiveness and purposiveness to organisms and their parts.

Arber’s suggestion for achieving a synthesis between these two positions drew on Spinoza’s way of resolving the ostensible antithesis between mind and body: taking them as two different aspects under which reality can be viewed. Similarly, she declared that mechanism and teleology “represent the organism under two different attributes—the physico-chemical attribute and the attribute of inherent directiveness” (Arber 1950, p. 208). Although she called her idea a “synthesis” and honored the achievements of both positions for biological research, Arber’s main aim was to promote the teleological standpoint. She was a defender of an organismal approach to biology and, in a time of reductionist research agendas and a growing dominance of experimentally-oriented plant physiology, she needed to defend her approach against the prevailing dominance of mechanistic thinking in biology.

This notwithstanding, there are several indications in Arber’s works that she possessed a rather skeptical attitude towards teleological explanations in biology. In her 1925 book *Monocotyledons: A Morphological Study*, she discussed orchids that have elaborate flowers resembling bees and thus seem to be “‘adapted to cross pollination’ but apparently pollinate themselves instead” (Arber 1925, p. 197). Commenting on this phenomenon, Arber wrote:

It must, indeed, be admitted that the consideration of this flower opens up a series of difficult questions, but the perplexity aroused by it can only be described as “unparalleled,” if we are determined to explain everything as the result of adaptation. A drawback of the teleological standpoint is that it so often leads to tilting at windmills—encouraging elaborate attacks on problems which have no existence, except in the minds of those to whom all structure is directly purposeful. (Arber 1925, p. 197)

A similarly negative assessment of teleologists is found in this quotation from her book *The Gramineae*, which was published in 1934: “The physiological anatomy of grass leaves offers a wide field for research, as yet insufficiently explored. The subject has been liable to fall into the hands of teleologists, and thus to come to a dead end” (Arber 1934, p. 306). The first quotation ascribes to the “teleological standpoint” the determination “to explain everything as the result of adaptation.” There are several other passages in her work, also as late as 1950, that support the assessment that Arber understood teleological explanations as ones that drew on the notions of function and evolutionary adaptation.^19^ As early as 1920, Arber already expressed her aversion against such an adaptationist standpoint in a letter to D’Arcy Wentworth Thompson, saying:

Some time ago I tried to put it to you that Phyllotaxis was adaptational, and you replied—“I should be at a loss to disprove the hypothesis that the Moon was made of green cheese.” I must confess that this was a shock to me, for I had been brought up an orthodox Darwinian; but the 4 years of working at morphology which have passed over my head since, have convinced me that you were right. I have been gradually shelling off the adaptation point of view, and I now believe that, at least in the case of leaf-structure, which I am struggling with, we get a series of harmonics upon certain definite basal form-types, which are wholly inexplicable from the utility standpoint.^20^

How do these two differing attitudes towards teleology, arguing for its necessity in biological thinking vs. rejecting adaptational explanations, fit together? I want to propose that Arber indeed had two different notions of teleology, which she developed on the basis of Aristotle’s formal and final cause, but about which she had differing opinions. I call these notions *formal* and *final* teleology, respectively. Arber defined the final cause as “the purpose or end of the thing” and the formal cause as “the essence or essential nature of the thing” (Arber 1950, p. 199). When studying organic form, these two causes built for her the class of teleological causes which could be contrasted to the physico-chemical causes, comprising the material and the efficient causes. Arber attributed to both teleological causes an *immanence* in the organism,^21^ but whereas the formal cause was “immanent in the organism, considered as a discrete individual,” the final cause was “immanent in the organism, considered as a part of the Whole” (Arber 1950, p. 207).

I interpret this passage as meaning that to Arber the formal cause described the essence of the organism, which initiated development according to the organism’s innate nature, whereas the final cause acted on the organism embedded in its environment, that is “the Whole. “The changes initiated by the final cause were therefore connected to environmental influences and bring about adaptations and improvements in function. So, when Arber talked about “teleologists” and her problems with the “utility standpoint,” she was thinking about final teleology: ascribing functions to structures that come into contact with the environment of an organism and looking for the reason behind adaptational changes in environmental influences on the organism. But when she ardently advocated for including teleology in biological reasoning, this was related to her notion of formal teleology: ascribing the directiveness in the development of an organism, or a morphological structure, to its essence and seeing the reason for its form in the actualization of inner potentialities.

Having thus established Arber’s two notions of teleology, the following sections present and discuss two of her theories which illustrate her understanding of formal teleology and how it informed her morphological research. The first one consists in her “partial-shoot theory of the leaf,” which she developed for the first time in 1941 and explicated in greater detail in her 1950 book *The Natural Philosophy of Plant Form*. The second one consists in her theory of parallelism in evolution, which she proposed in her 1925 book *Monocotyledons: A Morphological Study* and continued to develop in later works. These theories are representative examples, but are not the only places in which formal teleology informed Arber’s work. In fact, it lay at the heart of Arber’s definition of morphology itself since she stated that “the business of morphology [is] to connect into one coherent whole all that may be held to belong to the intrinsic nature of a living being” (Arber 1950, p. 2). The intrinsic nature of a being is exactly what is expressed by Aristotle’s formal cause and, as the following examples will show, Arber interpreted the realization of this intrinsic nature in terms of teleology and purposiveness.

## Formal Teleology in the Partial-Shoot Theory of the Leaf

Arber’s partial-shoot theory of the leaf is characterized by Kirchoff as “one of the best known alternatives to the classical model of the leaf” (Kirchoff 2001a, p. 1203).^22^ The classical model, which is still today the standard framework in plant morphology, divides the plant into three basic and mutually exclusive categories: leaf, stem and root (Kirchoff 2001a, p. 1203). Arber, on the other hand, based her theory on the equally ancient concept of the *shoot* as a unit which included both stem and leaf. She claimed that “the leaf is a partial-shoot, revealing an inherent urge towards becoming a whole-shoot, but never actually attaining this goal, since radial symmetry, and the capacity for apical growth suffer inhibition” (Arber 1950, p. 133). This means that, in Arber’s view, (i) the leaf was no fundamental category in the morphology of vascular plants but one that was derived from the category of the shoot; (ii) leaves, while being parts of an organism, did have their own goals and potentialities that they strove towards fulfilling; and (iii) the potentialities of the leaves to develop whole-shoot characters were inhibited, which resulted in the development of the leaf morphology that we can readily observe.^23^

**Fig. 2:**
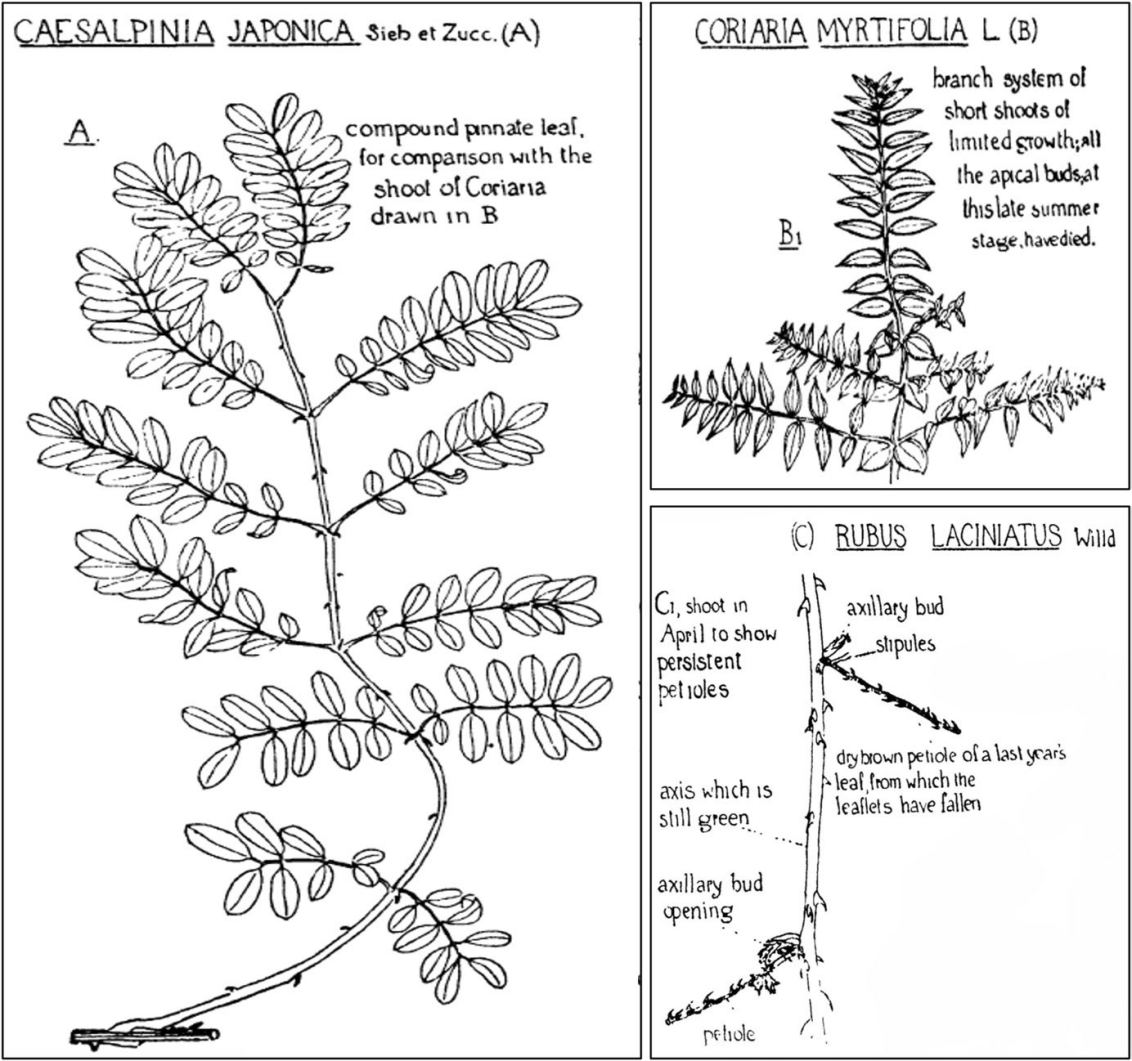
Arber’s view of plant morphology. Illustration, done by Agnes Arber, of a compound leaf in *Caesalpina japonica* (**A**), a branch system of short shoots in *Coriaria myrtifolia* (**B**), and the rachis of a compound leaf from which the leaflets have fallen in *Rubus laciniatus* (**C**). From Arber (1950, pp. 81, 90). Reproduced with permission of the Licensor through PLSclear

Why is the partial-shoot theory of the leaf an expression of formal teleology? Firstly, it dealt with a morphological problem—a problem pertaining to form. As stated above, morphology was for Arber a search for the intrinsic nature of an organism or its parts, with this “intrinsic nature” being the same thing that she called “essence” in her definition of the formal cause (see above). So, in this specific instance, she wanted to find out more about the intrinsic nature of the leaf. What she proposed was that the leaf is a partial shoot. She adopted this idea from Swiss botanist Casimir de Candolle (1836–1918), who spoke of the leaf as a “branch whose terminal cone is sterile” (de Candolle 1868, p. 50; author’s translation). In contrast to the classical model with the leaf as a basic category, Arber thus related the category of the leaf to that of the shoot, and highlighted their similarities. She characterized the leaf as “a reduced offspring, which partially repeats the characters of the shoot” (Arber 1950, p. 82), a tendency that could best be seen in compound leaves which reproduced the form of a complete shoot. This is illustrated in Fig. 2, wherein panel A depicts one single leaf of *Caesalpinia japonica*, and panel B shows a branch system of short shoots in *Coriaria myrtifolia*. The leaf in A is bipinnately compound, meaning that the lamina is divided twice: from the rachis (main axis of a compound leaf), several secondary axes branch off which bear small leaflets (technically *subleaflets*). These branches of secondary axes correspond to the lateral shoots in B that branch off from the main shoot. Furthermore, the similarities can even go as far as leaflets falling from the rachis, like leaves falling from a stem, shown in panel C of Fig. 2.

At the same time, Arber continued to draw a distinction between leaves and shoots by saying that leaves were only partial shoots: they were part of whole shoots and were not whole shoots themselves. This could be seen in their morphology. Contrary to typical whole shoots, most leaves did not exhibit radial symmetry and apical growth. Likewise, the inability of many leaves “to give rise either to partial or to whole-shoots” offered additional support to this view (Arber 1950, p. 105). But, and this was the second insight, leaves showed an “urge” to become whole shoots. Here, we clearly see the teleological component of Arber’s theory as she claimed that the leaf had a goal in its development. She spoke of a “latent whole-shoot character in the leaf” and of “potentialities” that the leaf endeavored to actualize (Arber 1950, p. 107). For this “urge,” Arber found evidence in the leaf through morphological and developmental observations. In early leaf development, there is an initial phase of apical growth producing a radial structure before the onset of the lamina development, which she interpreted as an effort of the embryonic leaf to develop whole-shoot characters. In mature leaves, trends towards “radialization” may also be found with leaves producing (for instance) additional wings, which make them approach the “radial character of the shoot” (Arber 1950, p. 107). However, although these efforts resulted in leaves of different species resembling whole shoots more or less closely, they never fully attained their goal. Arber neither attributed this inhibition to mechanisms within plant body organization, nor to external factors influencing developing leaves. Instead, that they never reached the status of a whole-shoot was for her something that was also grounded in the formal cause of the leaves: partialness lay in their “very essence” (Arber 1950, p. 78). Formal teleology thus not only invoked the actualization of potentialities, it also characterized the limits of development as set within the inner essence of an organ or an organism. Why exactly, from a physico-chemical point of view, the development of whole-shoot features died down so early in leaf development, Arber herself did not really know, calling it “one of the mysteries of ontogeny” (Arber 1941, p. 86).

Let us now move to the second example of formal teleology in action in Arber’s morphological work.

## Formal Teleology in the Theory of Parallelism in Evolution

Arber is not particularly known for discussing evolution or evolutionary theories. In fact, most treatments of her work characterize it as anti-evolutionist,^24^ and see this as one of the main features of Arber’s thinking that “put her outside mainstream botanical thought in her own time” (Flannery 2003, p. 281). However, this does not mean that Arber did not think about evolution or had nothing to say about it. In particular, her correspondences with Lady Isabel Browne and D’Arcy Wentworth Thompson give insights into her struggling with different evolutionary approaches and show that she was gradually becoming “profoundly sceptical about evolution.”^25^ Flannery ascribes Arber’s dislike for evolutionary theory to her disbelief in the idea that natural selection could explain all evolutionary changes (Flannery 2003).^26^ Born out of this skepticism about natural selection, Arber developed an alternative theory of evolution which drew on the concept of formal teleology. She stated:

[T]he occurrence of the same character in two forms of life—instead of being necessarily due to their “inheritance” of that character from some common ancestor who also possessed it—is often due to a tendency to parallel progression, which is naturally strongest in related forms, but which may also show itself in those which are systematically remote from one another. (Arber 1925, p. 223f)

The reason for this “parallel progression,” she saw in the inherent nature of the species. “It seems to me,” she wrote, “that the tendency to progress in a certain definite direction is as much an inherent character of a given race, as are the features of its chemistry or morphology” (Arber 1925, p. 231). This means that, instead of seeing similarities in the morphology of different species as an indication of close relationship and the possession of a recent common ancestor, we should attribute them to parallel developments caused by the innate nature of both species which led them to exhibit similar morphological forms. The phenomenon of certain forms that occur in closely, as well as in distantly related species had already been observed by different botanists; some of them had also already mentioned the idea of parallel developments in evolution. Arber referred in this regard to works by, for instance, Charles Naudin (1815–1899), Joseph Duval-Jouve (1810–1883), and Nikolai Ivanovic Vavilov (1887–1943), as well as her own morphological studies.

She showed, for example, that in four distinct cohorts within the monocotyledons, four “principal types of leaf recur,” constituting a “remarkable parallelism” (Arber 1925, pp. 228, 230). Instead of attributing these similarities to similar environmental influences that could have led to similar adaptations, which would be an argument based on final teleology, Arber argued from the stance of formal teleology and held that all changes due to environmental influences could only occur within a range of possibilities set by the species’ inherent nature. Taking the approach of formal teleology also made it easier to account for “the countless subtle differences between species that are difficult to attribute to natural selection,” to which Arber pointed repeatedly in her works (Flannery 1995, p. 546). For Arber, accepting the idea of the formal cause as a guiding principle in evolution meant that “we can no longer picture the evolution of plants, except as a journey along lines that are essentially fore-ordained” (Arber 1925, p. 232).

This deterministic view of evolution was an outcome of Arber’s adherence to formal teleology as well. It was the transmission of an idea about individual development to the level of the species. Just as the individual leaf on a plant had an urge to actualize its potentialities and realize its inner nature, species evolved along the lines that realized *their* inner nature. And just as the essence of the leaf also included its partialness, and thus the limits set for its development, the inherent nature of a species set the boundaries within which its morphology could develop. However, as in the case of the inhibition of whole-shoot characteristics in the leaf, Arber was eventually at a loss to explain the “great underlying principle, whatever it be, which determines these lines of progress,” stating that it was “entirely hidden from us” (Arber 1925, p. 232).

## Arber’s Ideas Then and Now

Putting these insights into a historical context, what the analysis of Arber’s ideas and convictions also shows is that she was a scholar who in a sense did not fully fit into her time. During her lifetime, biology turned its focus towards “laboratory disciplines” like biochemistry, genetics, and microbiology, while within botany, an analytically oriented plant physiology more and more dominated and overshadowed the fields of traditional plant anatomy and morphology (Jahn 1990, 2004; Nickelsen 2022). Arber, however, adhered to the old tradition of comparative morphology as well as to traditional methods and techniques. Furthermore, her whole conception of biological research was very much influenced by Goethe and the ideas of romantic biology, which can be seen, for instance, in the emphasis that she put on the visual, and the relation between science and art, in her continued search for scientific unity in diversity, and in her conviction that organisms should be studied and viewed as organic wholes. But in a time that she herself characterized as being focused on physico-chemical causes and the study of organisms by splitting them into ever smaller parts, Arber’s holistic approach isolated her from the larger mid-century trends of plant science in Great Britain, even though she continued to be highly regarded as a plant morphologist. On top of that came her anti-Darwinist tendencies which meant that “[a]t a time when the modern evolutionary synthesis was gaining ground, her approach obviously became less acceptable” (Flannery 2003, p. 285). This was also true for teleological reasonings within biology. Dresow and Love characterize the middle of the 20th century as a time of “teleophobia,” in which biology at large worked on distancing itself as far as possible from teleological accounts (Dresow and Love 2023; see also Baedke and Fábregas-Tejeda 2023).^27^

However, the analysis of her theories also can lead us to think that Arber was ahead of her time, as well, making her a possible source of inspiration for new anti-reductionist approaches to morphology and evolutionary biology.^28^ After a time in which the modern evolutionary synthesis dominated the thinking about evolution and the landscape of biological research and philosophy in general, it is “facing growing resistance from the scientific community today” (Rupik 2024, p. 10), allowing for the advancement of alternative approaches in evolutionary biology. For these approaches, Arber can be an inspiration because, through the analysis of her views on parallelism in evolution, we see that she was not simply anti-evolutionist. Instead, she wanted to draw attention to the idea that taking natural selection as the only factor relevant in evolutionary processes could result in an inadequate narrowing of scope and overlooking of other important factors.

This is exactly what the current proponents of a new “Extended Evolutionary Synthesis” are pointing to as well. They argue for the inclusion of the mechanisms of niche construction, developmental bias and inclusive inheritance in evolutionary theories and for adopting an organism-centered perspective which should incorporate the agency and self-organizational qualities of organisms into evolutionary theorizing (Laland et al. 2015; see also Sultan et al. 2022). This fits with the comeback of teleology during the last decade in the form of agency, which has become a much discussed topic in the philosophy of biology.^29^ Arber anticipated this development as well and argued for using both a mechanical *and* a teleological approach in the investigation of organismal change. Also, taking her views into account could inspire present-day philosophers of biology to take a closer look at the special characteristics of plants. While current approaches to teleology and agency mostly focus on the level of the whole organism, Arber pointed to the high autonomy of plant parts like leaves. This calls for new theories that consider the—possibly teleological—tensions and constraints in the relations of parts in the aggregative system of a plant.

Important supporting evidence for the view of the Extended Evolutionary Synthesis comes from the field of evolutionary developmental biology, or evo-devo, which seeks to integrate developmental and evolutionary biology, highlighting the importance of developmental processes for the evolutionary trajectories of species.^30^ Looking at Arber’s works, we find that some of her insights and approaches fit well within an evo-devo framework or, formulated differently, that evolutionary developmental biology tries to answer questions that Arber had previously formulated. Some contemporary insights from evo-devo seem to be the puzzle pieces that Arber was missing in her earlier reasoning. When she asked about the “great underlying principle (…) which determines these lines of progress” (Arber 1925, p. 232), she sought the mechanisms that guided the development of individual organisms, as well as whole species, and that caused the development of the same features in different species. This is one avenue of evolutionary developmental biology: determining the possibilities, set, for example, through gene-regulatory networks, of morphological development and variation, which are then targeted by natural selection. Studies on the genetic pathways responsible for phenotypic traits like flower symmetry or leaf shape now show that similar modifications in conserved gene families can lead to the independent development of the same morphological feature in different plant families, for example, bilateral flowers or compound leaves (see, for example, Preston et al. 2011). This mechanism seems to be a part of the “underlying principle” that Arber wanted to understand. One could even argue that evo-devo is the key to understanding formal teleology itself as it tries to uncover the mechanisms that guide the development of form in organisms. In fact, recent studies have already identified several genes that are involved in the orchestration of leaf growth and its termination, thus giving an insight into the mechanisms that Arber characterized as “one of the mysteries of ontogeny” (Arber 1941, p. 86; see also Minelli 2018, Chap. 5.2.1).

Further ways in which Arber’s formal teleology could be re-interpreted may prove themselves useful for present-day research. Examples for this can be found in the papers that came out of a symposium held in 1999 at the XVI International Botanical Congress in St. Louis, MO, USA, with the aim of celebrating the 50th anniversary of the publication of Arber’s book *The Natural Philosophy of Plant Form* in 2000, as well as her contributions to botany in general (Kirchoff 2001b, p. 1103). This symposium was organized by the botanists Bruce Kirchoff and Rolf Rutishauser under the title *From Agnes Arber to New Explanatory Models for Vascular Plant Development*. Seven papers that resulted from the symposium were published in the journal *Annals of Botany*—the journal in which Arber herself published most of her non-book publications. They include several examples for the application of some of Arber’s theories and ideas to current research in plant developmental biology (Barlow et al. 2001), morphology (Hofer et al. 2001; Rutishauser and Isler 2001), and phylogenetics (Kirchoff 2001a).

Especially in the field of morphology, Arber’s ideas already play an important role for alternative approaches in plant anatomy. One of these is even called “Fuzzy Arberian Morphology.” It follows Arber’s conception of the leaf and possibly also the root as a partial shoot and acknowledges that “structural categories and developmental processes conceivable in plants often have fuzzy borderlines (i.e. overlapping connotations)” (Rutishauser and Isler 2001, p. 1181). This idea, which is based on fuzzy set theory, can be helpful for interpreting developmental mosaics that can result in intermediate structures within the plant body or in “aberrant” plants. It is closely related to the concept that is known as “process morphology,” developed by plant morphologist Rolf Sattler, who attests to Arber having a “profound influence” on his life and scientific career (Sattler 2001, p. 1215). While the framework of Fuzzy Arberian Morphology still retains the idea of structural categories like leaf or root, process morphology replaces these categories by combinations of developmental processes. It reconceptualizes basic morphological assumptions by stating that “organisms are not structures that *have* developmental processes, they *are* developmental processes” (Rutishauser and Isler 2001, p. 1193f).^31^ This can be seen as a continuation of Arber’s dynamic outlook and the stress her partial-shoot theory laid upon the “dynamic study of relations and of parallels, rather than upon the static study of fixed types and absolute categories” (Arber 1950, p. 135). These interpretations are again very relevant for the field of plant evolution and development in the context of the development and identity of body parts in vascular plants and lead us to appreciate Arber’s considerable insights that have been long neglected.

## Conclusion

The central aim of this paper has been to focus on Agnes Arber’s special understanding of teleology in the natural world. An analysis of her writings shows that while Arber appeared skeptical towards final teleology, understood as adaptationist explanations of evolutionary changes in organisms that “aim” to fit environmental challenges, she held a very positive stance towards formal teleology, construed as inherent directiveness of organisms determining their form. Additionally, a closer look at two of her theories, namely the partial-shoot theory of the leaf and parallelism in evolution as a counter-proposal to natural selection, illustrates how Arber’s conceptions of morphology and evolution were informed by formal teleology and its implications. Putting her ideas into a broader context, I have suggested that while they might not have fit well into the scientific mainstream of her time, they may find fertile ground if considered from recent scientific developments. It might be timely to reconsider Arber’s work given that it appears to be directly relevant to particular areas of research, for example in evolution and development.

Arber’s theories did of course have their shortcomings. For example, she neglected, or at least downplayed, the influence of the environment on the development as well as the evolution of organisms. Her deterministic view of the evolutionary development of species therefore did not account for the profound influences that the environment could exert on the evolutionary trajectory of a species. But her insights were keen and there were many, especially when it came to her comprehensive account of teleology, that make Arber worthy of further study in the history and philosophy of the biological sciences, and that also may inspire contemporary plant scientists engaged in research. So, instead of seeing Arber “as a peripheral figure representative of outdated thinking in biology” (Flannery 2005, p. 274), we should rediscover her ideas and grant her the place that she deserves in the historiography of botany as well as the philosophy of biology.

## Data Availability

No datasets were generated or analysed during the current study.

## Footnotes

1 For discussions of the emergence of botany as a science during the eighteenth century, see Williams (2001), Smocovitis 2006a) and Sigrist (2013) and for accounts of the professionalization of botany, see, for example, Keeney (1992) and Secord (1994). Accounts of the suppression of female voices in botany can be found, for example, in Rudolph (1982, 1990), Shteir (1996), Henson (2002), and Hong (2021). Botany’s role in colonialism is discussed, for example, in Drayton (2000), Schiebinger (2004), Mickulas (2007), Endersby (2008), Miller and Reill (2011), Bleichmar (2012), and Subramaniam and Chatterjee (2024) and accounts of its influence on the development of the “evolutionary synthesis” can be found in Smocovitis 2006b, 2009, 2013) and Friedman and Diggle (2011).

2 Trewavas (2014) and Hiernaux (2023) both give some historical background in their treatments of plant intelligence and behavior. Historical treatments of the concept of plant sexuality can be found in Sandford (2023), and Subramaniam and Bartlett (2023).

3 For discussions of agency in plants, see, for example, Trewavas (2009), Ryan (2012), Gibson and Brits (2018), and Hiernaux (2021).

4 For a characterization of organism-centered biology and of organicism as one of the movements belonging to it, see Baedke (2019); see also Nicholson and Gawne (2015).

5 Arber is typically not counted among the main scholars of early twentieth century organicism (e.g., in Nicholson and Gawne 2015 and Baedke 2019) but she was clearly sympathetic to the organicist movement and saw it as the best way to resolve the opposition between vitalism and mechanism (Arber 1964). In their analyses, both Bruce (2014) and Yilmaz (2024) characterize her as an organicist thinker.

6 Other obituaries include Stearn (1960), Thomas (1960a) and Corner (1961).

7 Publications that focus on specific books by Arber include Flannery (2003), who discusses *The Mind and the Eye* as well as *The Natural Philosophy of Plant Form*, and Feola (2019), who focuses on *Herbals: Their Origin and Evolution* and *The Mind and the Eye*. Comparisons between Arber and other scholars of plant morphology are drawn in Flannery (1995) who compares Arber and Goethe, as well as Claßen-Bockhoff (2001), who writes on Arber, Wilhelm Troll and Walter Zimmermann. Applications of Arber’s theories to current research in plant sciences can be found in Barlow et al. (2001), Hofer et al. (2001), Kirchoff (2001a) and Rutishauser and Isler (2001).

8 The archival material on Agnes Arber at the Hunt Institute for Botanical Documentation includes a handmade herbarium with dried plants and handwritten names in it. The first page is marked with “Agnes aged seven. 4th May 1886,” Box 2, Folder An 1, Agnes Robertson Arber (1879–1960) papers 1886–1985, Hunt Institute for Botanical Documentation, Carnegie Mellon University, Pittsburgh, Pennsylvania, USA, accessed November 30, 2023, https://www.huntbotanical.org/archives/detail.php?16. Heretofore abbreviated as “Arber Papers, 1886–1985”.

9 Flannery reports that Arber borrowed a microtome and microscopes from the Balfour Laboratory and probably had no additional equipment. Furthermore, she had no electricity at her home (Flannery 2003).

10 “[M]y father, who is an artist, is an enthusist [sic] about ‘learning to draw is learning to see’ and he gave us each a daily lesson, before he went to the studio, from the time we were three until we went to school.” Arber to D’Arcy Wentworth Thompson, October 10, 1920, Box 1, Folder 5, Arber Papers 1886–1985.

11 Based on the description of the intended drawing that Thompson gave in his letter to Arber from 7 August 1941, it is probably Fig. 485 on p. 1012, which shows a “rough paper model of a fowl’s pelvis” (Thompson 1945, p. 1012). It is not possible, however, to verify if this drawing was really made by Arber because there is no indication of the illustrator.

12 See Corner (1961).

13 Archival material at the Hunt Institute for Botanical Documentation includes several boxes of correspondence.

14 Arber to B. C. Sharman, July 25, 1939, Box 1, Folder 3, Arber Papers 1886–1985.

15 Arber to D’Arcy Wentworth Thompson, November 20, 1932, Papers of D’Arcy Wentworth Thompson, item ms20103, University of St. Andrews, Scotland.

16 Arber to D’Arcy Wentworth Thompson, November 20, 1932, Papers of D’Arcy Wentworth Thompson, item ms20103, University of St. Andrews, Scotland.

17 This striving for creating synthesis out of seemingly opposed theories played an important role in Arber’s theoretical work and appeared as a guiding principle in several places throughout this and other books. See, for instance, the discussion of the partial-shoot theory of the leaf later in this paper or Chaps. 8 and 9 of Arber (1964). Additionally, Flannery points to Arber’s last book, *The Manifold and the One*, which shows “the depth of her dedication to the concept of oneness” and “helps to explain why she is so committed to finding a basis for underlying unity in plant form and for reconciling seemingly opposite viewpoints—major themes in her previous two books” (Flannery 2003, p. 282).

18 Arber dealt with Troll’s theories and methods in many of her written works. Similar to her attitude towards Goethe, she held many of his views in high esteem and called his work and school “the principle factor in the development of modern morphological botany” (Arber 1941, p. 83), but also engaged critically with his approaches saying, for example, that he presents his ideas upon an “somewhat confusing background” (Arber 1937, p. 179) and that “if one adopts his synthetic and holistic attitude so completely as to exclude analysis, scientific work, in the strict sense, comes to a full stop” (Arber 1933, p. 239). In a letter from 1939, Troll addressed Arber as a “kindred spirit” and appreciated her “deep insight into the character of morphological thinking” (Wilhelm Troll to Arber, May 23, 1939, Box 1, Folder 3, Arber Papers 1886–1985; author’s translation).

19 In *The Natural Philosophy of Plant Form*, for instance, Arber illustrated the difference between the mechanical and the teleological standpoint by drawing on the example of explaining “the flattened and expanded character of the green leaf,” saying that a teleologist “would say that the dorsiventrality and expansion of the leaf have been developed in order to provide an effective assimilating, breathing, and transpiring organ” (Arber 1950, p. 200). This shows that, for Arber, explaining a biological structure teleologically meant to give an evolutionary explanation regarding the supposed purpose that the structure had for the organism. Other examples include her characterization of the botanists Federico Delpino and Wilhelm Troll, of whom she said that “Delpino was essentially a *teleologist*, whereas Troll rejects *adaptational explanations*” (Arber 1950, p. 155; emphasis added), and a discussion of the development of vascular bundles in axis and leaves, where she wrote: “Attempts have been made to read an *adaptational meaning* into the outward curvature of the basal region of such leaf-traces” and later: “But any such *teleological* view is ruled out of court, when we realise that this outward curvature is a purely automatic result of the massive conical form of the procambial layer” (Arber 1925, p. 37; emphasis added).

20 Arber to D’Arcy Wentworth Thompson, November 5, 1920, Box 1, Folder 5, Arber Papers 1886–1985.

21 Declaring both causes as immanent, that is intrinsically belonging to organisms, precluded any theological implications commonly associated with *external teleology* (for discussion, see Toepfer 2004). When the teleological causes originated in the organisms themselves, teleology could no longer be connected to the idea of a God or designer who created organisms and their parts as they are to fulfill a certain purpose, or to the idea that certain species are an end for others, like bees having the purpose to pollinate flowers or plants bearing fruit to provide food for humans.

22 Kirchoff also argues that it is not a theory in the modern sense but rather “a summary of empirical observations” that Arber put forward to “help us see the plant with fresh eyes” (Kirchoff 2001a, p. 1204).

23 Furthermore, through characterizing the leaf as a partial-shoot and thus taking the shoot as the basic category of plant morphology, but at the same time acknowledging the discontinuity between stem and leaf, Arber created a synthesis between the stem-and-leaf-hypothesis of the classical model and the shoot-hypothesis of the alternative model, showing again her striving for unity.

24 See, for example, Tansley (1952), Eyde (1975), or Feola (2019).

25 “I suppose that what it really comes to is that I am becoming profoundly sceptical about evolution; I feel at any rate that there is something radically wrong in the way we look at the process that goes by that name—but what that something is I do not know and would give much to know.” Arber to Lady Isabel Browne, August 11, 1941, Box 1, Folder 1, Arber Papers 1886–1985.

26 In *The Mind and the Eye*, Arber made this clear: “[W]e might claim with justice that *if* Natural Selection has governed the past development of the organic world, animals and plants to-day will show adaptations to their environments. Further we may observe, rightly, that animals and plants *are* adapted to their environments. It is not justifiable, however, to conclude from these two statements that Natural Selection has, as a matter of history, controlled the development of the organic realm. All we can say is that Natural Selection is one of the antecedent factors which *might* have produced this effect; but our reasoning gives no guarantee that this effect has not been, in fact, brought about by other causes, perhaps of a totally different kind” (Arber 1964, p. 24; emphases in the original).

27 As pointed out in the introduction, vitalists and organicists defended teleological ideas during this time. These camps were, however, minorities within the fields of philosophy and biology and “remained outside the biological mainstream” which meant that their work “failed to penetrate very far into the main currents of biological thought” (Dresow and Love 2023, p. 104). Also, approaching 1950, most activity around vitalist and organicist accounts had stopped (Baedke 2019).

28 See also Flannery (2003) for a similar argument.

29 For recent monographs and edited volumes on the topic, see, for example, Walsh (2015), Okasha (2018), and Fábregas-Tejeda et al. (2024).

30 For introductions to the field of evolutionary developmental biology, see, for example, Gilbert and Epel (2009), Minelli (2009), and Nuño De La Rosa and Müller (2021).

31 For more information on Fuzzy Arberian Morphology and process morphology, see also Rutishauser (1999), Kirchoff et al. (2008), Rutishauser (2020), Sattler and Rutishauser (2022).
